# The Synaptic Vesicle Priming Protein Munc13 Mediates Evoked Somatodendritic Dopamine Release

**DOI:** 10.1523/JNEUROSCI.2320-25.2026

**Published:** 2026-06-26

**Authors:** Joseph J. Lebowitz, Aditi Banerjee, Gillian Handy, John T. Williams, Pascal S. Kaeser

**Affiliations:** ^1^Vollum Institute, Oregon Health and Science University, Portland, Oregon, 97239; ^2^Department of Neurobiology, Harvard Medical School, Boston, Massachusetts, 02115

**Keywords:** active zone, dopamine, Munc13, secretion, somatodendritic release, spontaneous release

## Abstract

Midbrain dopamine neurons release dopamine not only from their axons but also from their somata and dendrites. Shared and distinct properties have been proposed for somatodendritic and axonal release, but the mechanisms of somatodendritic release remain unclear. We here used gene knock-out, electrophysiology, and imaging to define roles of the synaptic vesicle priming protein Munc13 in somatodendritic dopamine release in comparison with axonal secretion. We characterized mice of either sex and found that Munc13 ablation decreased evoked but not spontaneous somatodendritic dopamine transmission measured as D2 receptor-mediated currents. Imaging with a fluorescent sensor confirmed the importance of Munc13 in evoked somatodendritic and axonal dopamine secretion. Pharmacological experiments revealed a modest contribution of release from norepinephrine axons to D2 receptor-mediated currents, and the relative contribution was enhanced after Munc13 knock-out. Altogether, these data establish important roles of Munc13 in evoked somatodendritic release. These roles are similar to Munc13 functions in axonal dopamine release and at fast synapses. Spontaneous midbrain dopamine release was not impaired by Munc13 ablation from dopamine neurons and may rely on a release pathway that is independent of the prototypical release machinery employed at synapses.

## Significance Statement

Neurons release neurotransmitter with high spatiotemporal precision from axonal nerve terminals. In addition, neurons also secrete transmitters such as monoamines, neurotrophins, and neuropeptides from their somata and dendrites. While axonal release has been well characterized, less is known about mechanisms of somatodendritic secretion. The presented data establish that the synaptic vesicle priming protein Munc13 is important for evoked somatodendritic dopamine release. In contrast, spontaneous somatodendritic dopamine release is not affected by Munc13 ablation from dopamine neurons in the tested mouse mutants. The Munc13 dependence of evoked somatodendritic secretion supports the model of spatiotemporally precise signaling for this form of transmission. Spontaneous dopamine release may rely on a pathway that is independent of the classical neurotransmitter release machinery.

## Introduction

Neurotransmitter release from axon terminals is mediated by specialized secretory machinery that enables spatiotemporally precise vesicle fusion (Südhof, 2012; Kaeser and Regehr, 2014). Most neurons also release neurotransmitters from the somatodendritic compartment, and both synaptic point-to-point transmission and volume transmission that lacks synaptic structure may mediate this dendro-dendritic signaling (Kennedy and Ehlers, 2011). Midbrain dopamine neurons, for example, release dopamine from somata and dendrites, and D2 receptor activation inhibits their firing (Björklund and Lindvall, 1975; Geffen et al., 1976; Aghajanian and Bunney, 1977; Araneda and Bustos, 1989; Pucak and Grace, 1994).

Recent work identified molecular machinery for somatodendritic dopamine release. First studies established that release is vesicular and relies on SNARE proteins for exocytosis (Bergquist et al., 2002; Beckstead et al., 2004; Fortin et al., 2006). Evoked somatodendritic release is Ca^2+^-dependent and utilizes Synaptotagmin-1 as a Ca^2+^ sensor (Beckstead et al., 2004; Lebowitz et al., 2023), and additional Ca^2+^ sensors might regulate it (Mendez et al., 2011; Delignat-Lavaud et al., 2022; Hikima et al., 2022; Lebowitz et al., 2024). Voltage-gated Ca^2+^ channels are a Ca^2+^ source, though the identity of the channels remains uncertain (Beckstead et al., 2004; Mendez et al., 2011). Furthermore, the active-zone protein RIM is important for stimulus-induced somatodendritic dopamine release, suggesting that preorganized protein complexes tethered by RIM mediate these fusion events similar to axonal dopamine secretion (Liu et al., 2018; Robinson et al., 2019). In aggregate, these studies revealed that the molecular machinery for somatodendritic dopamine release resembles that of classical synaptic release, supporting the model that small, clear vesicles might be the fusing membrane compartment (Liu et al., 2021; Özçete et al., 2024).

Spontaneous somatodendritic dopamine release occurs in the absence of action potentials, is Ca^2+^-independent, and does not necessitate RIM or Synaptotagmin-1 in dopamine neurons (Gantz et al., 2013; Robinson et al., 2019; Lebowitz et al., 2023). While detected in somatodendritic regions as miniature events recorded as D2 receptor-mediated inhibitory postsynaptic currents (D2-IPSCs), it has remained uncertain whether this form of release is also present in axons. Ablating RIM from dopamine neurons does not remove all axonal release, striatal microdialysis detects activity-independent extracellular dopamine, and nanofilm sensors also detect activity-independent axonal release in cultured dopamine neurons (Liu et al., 2018; Banerjee et al., 2020; Bulumulla et al., 2022). This suggests that spontaneous dopamine release occurs in axons. Altogether, evoked and spontaneous dopamine release from axons, somata, and dendrites may all contribute to dopamine function and to dopamine-associated disorders (Gantz et al., 2013; Rice and Patel, 2015; Robinson et al., 2019; González-Rodríguez et al., 2021; Liu et al., 2021; Cai et al., 2024; Salvatore, 2024).

Here, we assessed roles of Munc13 in somatodendritic dopamine release. Munc13 is important for axonal release across species and cell types through its activities as a synaptic vesicle priming protein (Aravamudan et al., 1999; Augustin et al., 1999; Richmond et al., 1999; Varoqueaux et al., 2002; Chen et al., 2013; Imig et al., 2014; Banerjee et al., 2022; Elizarova et al., 2022; Tan et al., 2022a,b). In mice with Munc13 ablation in dopamine neurons, we found a strong reduction in evoked somatodendritic dopamine release measured as D2-IPSCs. Dopamine release examined with a fluorescent sensor revealed a similar disruption when comparing somatodendritic release in the midbrain with axonal release in the striatum. Spontaneous dopamine transmission in the midbrain was unaffected by Munc13 ablation, bolstering the model of a separate secretory pathway. Finally, a small component of the midbrain D2-IPSC was due to norepinephrine innervation, reminiscent of the observation that D2 receptor-based dopamine sensors can report norepinephrine (López et al., 2026). The relative norepinephrine contribution to D2-IPSCs was increased after Munc13 ablation. Overall, these findings establish that Munc13 mediates evoked somatodendritic dopamine release. Our work supports that mechanisms for evoked somatodendritic release resemble those for axonal transmitter secretion and is suggestive of preassembled release sites for efficient and temporally precise dopamine signaling.

## Materials and Methods

### Mice

Munc13 cKO^DA^ mice were crossed as described before (Banerjee et al., 2022). Conditional Munc13-1 floxed mice (MGI:7276178; Banerjee et al., 2022) were crossed to constitutive Munc13-2 knock-out mice (MGI:2449706; Varoqueaux et al., 2002), constitutive Munc13-3 knock-out mice (MGI:2449467; Augustin et al., 2001), and DAT^IRES-cre^ mice (JAX: 006660; Backman et al., 2006). Munc13 cKO^DA^ mice were homozygote for Munc13-1 floxed, Munc13-2 null, and Munc13-3 null and heterozygote for DAT^IRES-cre^. Munc13 control mice were heterozygote for Munc13-1 floxed and Munc13-2 null, homozygote for Munc13-3 null, and heterozygote for DAT^IRES-Cre^. We used littermate or age-matched mice of either sex from the same breeding colony for experiments. Experiments in Figures S1 and S2 were conducted in mice of either sex heterozygote for DAT^IRES-cre^. Mice were genotyped in the lab or by Transnetyx. Experiments were performed in accordance with protocols approved by Animal Care and Use Committees at Harvard Medical School and Oregon Health and Science University.

### Stereotaxic surgeries

D2 receptor or GRAB_DA_ were delivered via stereotaxic injections of AAVs into the mouse brain. AAVs for D2 receptor expression were custom made as described (Lebowitz et al., 2023), and AAVs for GRAB_DA_ expression were purchased. For AAV9-GRAB_DA2m_ [also called AAV-hSyn-GRABDA2m, rAAV-hSyn-DA4.4; serotype, AAV2/9; Biohippo BHV12400441-9 (Sun et al., 2020)], 36–67-d-old mice were anesthetized with isoflurane and mounted on a stereotaxic frame. Anesthesia was maintained throughout surgery using isoflurane. After exposure of the skull, a burr hole was drilled for a unilateral AAV injection. Next, 1 µl of AAV (1–2 × 10^12^ viral genomic copies/ml) was injected into the dorsal striatum a rate of 0.1 µl/min using a microinjection syringe pump, coordinates were 1.0 mm anterior to the bregma, 2.0 mm lateral, and 2.5 mm below pia. Analgesia was provided following approved protocols. Experiments were conducted 48–60 d following injection. For AAV-GRAB-DA_3m_ [also called rAAV-hSyn-DA3m-WPRE-PA; serotype, AAV2/RETRO, BioHippo BHV12400547-12 (Zhuo et al., 2024)], 70–150-d-old mice were anesthetized with isoflurane and mounted on a stereotaxic frame. Anesthesia was maintained throughout surgery using isoflurane. After exposure of the skull, two burr holes were drilled for bilateral AAV injections. On both sides, 120 nl of AAV (titer, 5.0 × 10^12^ viral genomic copies/ml) were injected into the striatum at each of three locations along the dorsal/ventral axis. Virus was injected at a rate of 0.3 µl/min using a microinjection syringe pump, coordinates were 1.25 mm anterior to the bregma, 1.50 mm lateral, and 3.40, 3.30, and 3.20 mm below pia. Analgesia was provided following approved protocols. Experiments were conducted 20–45 d following injection. For AAV-hD2Rs-P2A-GFP [custom made from pFB-AAV9-CAG-hD2Rshort-SNAP-p2A-GFP-WPRE-SV40pA, lab plasmid code p1038 (Lebowitz et al., 2023)], 90–140-d-old mice were anesthetized with isoflurane and mounted on a stereotaxic frame. Anesthesia was maintained throughout surgery using isoflurane. After exposure of the skull, two burr holes were drilled for bilateral AAV injections. On each side, 200 nl of AAV (titer, 2.15 × 10^13^ viral genomic copies/ml) were injected into the midbrain. Virus was injected at a rate of 0.3 µl/min using a microinjection syringe pump. Coordinates were 2.3 mm posterior to the bregma, 1.3 mm lateral, and 4.5 mm below the pia. Postoperative analgesia was provided following approved protocols. Experiments were conducted 14–21 d following injection.

### Brain slice preparation

For D2-IPSC recordings and GRAB_DA3m_ imaging, mice (110–209 d old) were deeply anesthetized with isoflurane and decapitated. The brain was dissected into warm (32–35°C) Krebs buffer containing the following (in mM): 126 NaCl, 2.5 KCl, 1.2 MgCl_2_, 2.4 CaCl_2_, 1.4 NaH_2_PO_4_, 25 NaHCO_3_, and 11 dextrose. Krebs buffer was bubbled with 95% O_2_/5% CO_2_, pH 7.4, prior to decapitation and continuously throughout cutting and recovery and contained 10 µM MK-801. Horizontal brain slices containing the ventral midbrain were prepared at a thickness of 222 µm on a vibrating microtome and allowed to recover for at least 30 min at 30–32°C prior to recording or imaging. For GRAB_DA2m_ imaging, mice (84–127 d old) were deeply anesthetized with isoflurane and decapitated. Brains were dissected out, and 250-µm-thick parasagittal sections of the striatum were cut on a vibrating microtome in ice-cold cutting solution containing the following (in mM): 7.5 MgSO_4_, 75 sucrose, 75 NaCl, 1 NaH_2_PO_4_, 12 glucose, 26.2 NaHCO_3_, 2.5 KCl, 1 sodium ascorbate, 1 myo-inositol, and 3 sodium pyruvate bubbled with 95% O_2_/5% CO_2_, pH 7.4, 300–305 mOsm. Slices were incubated in recovery solution for at least 1 h at room temperature (21–25°C) containing the following (in mM): 126 NaCl, 1 NaH_2_PO_4_, 2.5 KCl, 2 CaCl_2_, 12 glucose, 1.3 MgSO4, 12 glucose, 26.2 NaHCO3, 1 sodium ascorbate, 1 myo-inositol, and 3 sodium pyruvate, bubbled with 95% O_2_/5% CO_2_, pH 7.4, 300–305 mOsm.

### Electrophysiology

Experiments were performed using previously established methodology (Beckstead et al., 2004; Gantz et al., 2013; Robinson et al., 2019; Lebowitz et al., 2023). Slices were bisected along the midline, and experiments were carried out in hemisections placed in a recording chamber under continuous perfusion with Krebs buffer (2–3 ml/min) at 34°C bubbled with 95% O_2_/5% CO_2_, pH 7.4. Krebs buffer for recording also contained NBQX (600 nM), CGP55845 (300 nM), and picrotoxin (100 µM). In hemisected horizontal midbrain slices, dopamine neurons in the substantia nigra pars compacta (SNc) were identified by size, morphology, and position lateral to the medial terminal nucleus of the accessory optic system. Recordings were made using glass pipettes (initial resistance, 1.2–1.6 MΩ) filled with an internal solution containing the following (in mM): 100 K-methanesulfonate, 20 NaCl, 1.5 MgCl_2_, 10 HEPES (K), 2 ATP, 0.3 GTP, 10 phosphocreatine, and 10 BAPTA (4 K), pH 7.4, 270–290 mOsm. Firing rates were recorded for ≥1 min in cell-attached mode prior to break-in, and action potentials were identified using an amplitude threshold. The holding potential was set to −55 mV prior to break-in, and cell capacitance, membrane resistance, and series resistance were measured with a 5 mV test pulse immediately after break-in. The uncompensated series resistance was monitored, and cells with an initial series resistance ≥10 MΩ were excluded from experiments. The *I_h_* current was measured as the average amplitude over the last 25 ms of a 2 s, −50 mV hyperpolarizing voltage step. D2-IPSCs were recorded no sooner than 5 min after break-in. A monopolar electrode was positioned ∼75–100 µm caudal to the recorded cell, and D2-IPSCs were induced with a train of five stimuli at 40 Hz using 50, 150, 250, and 350 µA stimulus intensities. The order of stimulation (ascending or descending) was alternated between cells. Each intensity was tested with three to five consecutive sweeps with an intersweep interval of 1 min, and the average amplitude of those sweeps was quantified at each stimulus intensity. Series resistance was monitored between different stimulus intensities and cells with a series resistance that exceeded 10 MΩ were discarded. The sensitivity to voltage-gated Na^+^ channel blockade was examined by comparing the D2-IPSC amplitude evoked with a 350 µA stimulus before and after the addition of 1 µM tetrodotoxin (TTX). Spontaneous D2-IPSCs were measured over a 5 min period starting no sooner than 10 min after break-in and detected automatically using a sliding template in Axograph (RRID:SCR_014284). In a second line of analyses, manual identification of spontaneous D2-IPSCs by an experimenter blind to genotype yielded results that were similar to those measured with the template function and shown in Figure 4, *C* and *D*. The sensitivity of D2-IPSCs to D2 receptor or voltage-gated Ca^2+^ channel blockade was assessed before and after addition of 1 µM sulpiride (Fig. S1) or 300 µM Cd^2+^ (Fig. S2), respectively; the sliding template method was used for detection of spontaneous events. If no IPSCs were detected, the frequency was considered 0 Hz. Occasionally, for the last cell of a given slice for the experiments in Figure 4, sulpiride (1 µM) was added at the end of the recording to qualitatively evaluate the dependence of spontaneous D2-IPSCs on D2 receptor activation and sulpiride blocked spontaneous events. D2 receptor activation by exogenous dopamine was measured by superfusing dopamine (1 or 10 µM) and is reported as the maximum change in current density. First, 1 µM dopamine was superfused until a peak current was attained (for ∼2 min) and then removed. After the current returned to baseline, 10 µM dopamine was superfused until a new peak was attained (for ∼2 min). Current density was calculated by dividing the amplitude of the change in holding current by the recorded cell's capacitance. Norepinephrine axon contribution was estimated by sequential perfusion of UK14,304 (1 µM) followed by idazoxan (1 µM). Effects were calculated using the final three sweeps (at 3–5 min) during an 8 min perfusion for each condition. Representative traces of evoked D2-IPSCs are the average of three consecutive sweeps. Representative traces of pacemaker firing, dopamine superfusion, and spontaneous D2-IPSCs are from single sweeps.

### Widefield imaging in the striatum

GRAB_DA2m_ imaging in parasagittal slices containing the dorsal striatum was performed following established methodology (Liu et al., 2022; Cai et al., 2024) with modifications. Images were acquired with a widefield fluorescence microscope (Olympus BX51) with a 470 nm excitation LED, a 4× objective, and a scientific metal–oxide–semiconductor camera (Hamamatsu ORCA-Flash4.0) in artificial cerebrospinal fluid (ACSF) containing the following (in mM): 126 NaCl, 26.2 NaHCO_3_, 2.5 KCl, 1.3 MgSO_4_, 2 CaCl_2_, 1 NaH_2_PO_4_, and 12 glucose. The ACSF was heated to 34–36°C, and slices were perfused with a flow rate of 2–3 ml/min, and 1 µM DhβE was included in the bath. Solutions were continuously bubbled with 95% O_2_/5% CO_2_, and recordings were completed within 5 h of slicing. Electrical stimulation was delivered with an intensity of 90 µA (biphasic wave, 0.25 ms in each phase) and with a linear stimulus isolator (A395, World Precision Instruments) through a unipolar glass pipette (tip diameter of 2–4 µm) filled with ACSF, using single pulses or 10 pulse stimulus trains at 10 Hz. Image acquisition, LED illumination, and electrical stimulation were controlled with a digitizer (Molecular Devices, Digidata 1440A). LED power was set to 10% across genotypes and repeats. Images were acquired at 512 × 512 pixels per frame, with a sampling frequency of 50 fps and an exposure time of 20 ms per frame. Fields of view were selected based on the presence of similar visible baseline GRAB_DA_ fluorescence prior to stimulation. Three sweeps were performed and averaged for each area for single stimuli and for 10 stimuli at 10 Hz. A single pixel represents a striatal area of 6.4 × 6.4 µm^2^. For analyses, ImageJ/Fiji (RRID:SCR_002285; Schindelin et al., 2012) was used, and background fluorescence was estimated from regions of no sensor expression and subtracted from each image frame. Pixels with intensity values in the top 50% of the intensity histogram of the image stack generated from background subtracted images were used for plotting the Δ*F*/*F*_0_ time course. *F*_0_ was calculated as the average GRAB_DA2m_ signal over 0.5 s (25 frames) immediately before stimulation (*F*_0_ as mean ± SEM in Munc13 control, 696.2 ± 92.7 arbitrary fluorescence units; 12 images of 12 slices from 3 mice; Munc13 cKO^DA^, 974.2 ± 131.7; 12/12/3; *t*_(22)_ = 1.726; *p* = 0.09; unpaired *t* test). Δ*F*/*F*_0_ was calculated for each pixel and quantified. For the peak plots, the maximum value of Δ*F*/*F*_0_ within 0.7 or 1.1 s for single or train stimuli, respectively, was plotted. Sample heatmaps show the peak Δ*F*/*F*_0_ image frame after background subtraction and with brightness and contrast adjustments identical across genotypes; the display color range was set to illustrate the full range of the Δ*F*/*F*_0_ signal, and the underlying raw Δ*F*/*F*_0_ was not saturated.

### Confocal imaging in the striatum and midbrain

GRAB_DA3m_ imaging on hemisected horizontal slices was carried following methodology described before (Rodriguez-Contreras et al., 2025) but with an upright microscope (Olympus BX51 WI), a 60× objective (Olympus LUMPlanFL 60X/1.00W), and a Yokogawa CSU10-B-F300-E-B488 spinning-disc confocal unit. Excitation was at 488 nm with a Cobolt 06-MLD laser controlled by a NEOS AOTF module. Fluorescence was detected and recorded at 10 Hz with a Photometrics Prime 95B camera. Hardware and acquisition settings were controlled by µManager2.0 (RRID:SCR_000415). Stimulation was carried out using Axograph and triggered by µManager2.0 at the start of each video to synchronize acquisition and stimulus timing. Stimulation was delivered with a monopolar electrode as described for evoked D2-IPSCs at 250 µA stimulation intensity, and five pulses at 40 Hz were applied. Fields of view were selected based on the presence of similar visible baseline GRAB_DA_ fluorescence prior to stimulation. Each field was imaged once, and 3–7 images were captured from each slice. The duration between stimuli was ≥5 min. For analyses, a maximum intensity projection image was generated, and the pixels with intensities in the top 75% in the intensity histogram of the image stack were used to generate a region of interest (ROI) in ImageJ/Fiji. GRAB_DA3m_ intensity was measured as the average intensity in the ROI. *F*_0_ was defined as the average fluorescence over 2 s (20 frames) preceding stimulation [*F*_0_ as mean ± SEM in striatum; Munc13 control, 126.3 ± 9.7 arbitrary fluorescence units; six fields of view of four slices from four mice; Munc13 cKO^DA^, 111.4 ± 0.3; 7/3/3; *U*(*n*_1_ = 6; *n*_2_ = 7) = 11; *p* = 0.18; Mann–Whitney rank sum test; *F*_0_ in midbrain; Munc13 control, 114.3 ± 0.6; 26/6/3; Munc13 cKO^DA^, 124.9 ± 2.2; 14/6/4; *U*(*n*_1_ = 26; *n*_2_ = 14) = 57; *p* < 0.01; Mann–Whitney rank sum test] and used to determine Δ*F*/*F*_0_. The peak fluorescence value was extracted as the maximum change in Δ*F*/*F*_0_ over the same ROI from a single image frame from the time series. Sample heatmaps show the peak Δ*F*/*F*_0_ image frame with brightness and contrast adjustments identical across genotypes and brain regions; the display color range was set to illustrate the full range of Δ*F*/*F*_0_ signal, and the underlying raw Δ*F*/*F*_0_ was not saturated.

### Experimental design and statistical analyses

Summary data are presented as mean ± SEM with individual values shown as circles. Statistical significance is denoted as *p < 0.05, **p < 0.01, and ***p < 0.001 in each figure. Statistical testing was done in GraphPad Prism 10 (RRID:SCR_002798). The number of observations is described in each figure legend. Numerical values for the data presented in figures were deposited at 10.5281/zenodo.20067463. For comparison of two groups, datasets were tested for normality with a Shapiro–Wilk test. Normally distributed data were assessed with paired or unpaired Student's *t* tests, and non-normally distributed data with Mann–Whitney (unpaired) or Wilcoxon matched-pairs (paired) rank sum tests. For comparisons of multiple groups and variables, two-way ANOVA was used with individual comparisons made using the post hoc test listed in the corresponding figure legend. Experiments with genotype comparisons were performed by an experimenter blind to genotype during data acquisition and analyses.

## Results

### Munc13 ablation disrupts evoked somatodendritic dopamine release

**Figure 1.**
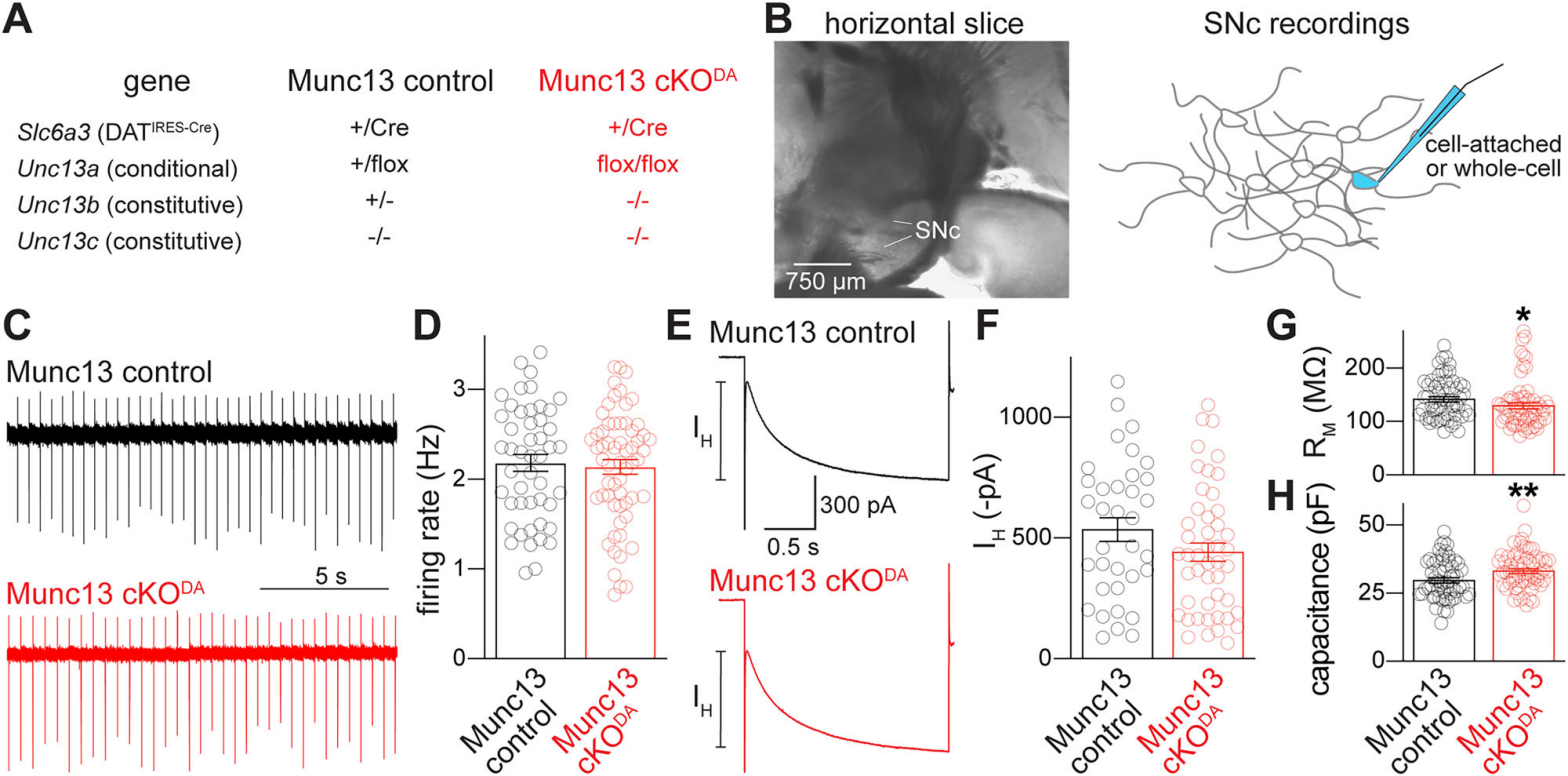
Electrical properties of dopamine neurons after Munc13 ablation. ***A***, Genetic strategy for Munc13 ablation as established before (Banerjee et al., 2022); Munc13-1 ablation is dopamine neuron-specific; Munc13-2 and Munc13-3 ablations are constitutive. ***B***, Bright-field image of a horizontal brain slice and a schematic of the recordings in the SNc. ***C*, *D***, Example traces (***C***) and quantification of rates (***D***) of spontaneous firing of dopamine neurons, measured cell-attached over 2–4 min prior to break-in; Munc13 control 48 cells from 34 slices of 18 mice, Munc13 cKO^DA^ 60/40/21. ***E*, *F***, Example traces (***E***) and quantification of the amplitude (***F***) of HCN-mediated inward currents (*I_H_*) in response to a −50 mV, 2 s hyperpolarizing step; Munc13 control 35/25/15, Munc13 cKO^DA^ 47/32/15. ***G*, *H***, Quantification of the input resistance (***G***) and cell capacitance (***H***) measured after break-in; Munc13 control 57/33/16, Munc13 cKO^DA^ 64/45/15. Data are shown as mean ± SEM; *p < 0.05; **p < 0.01; assessed by unpaired *t* tests (***D***, *t*_(106)_ = 0.3527; *p* = 0.725; ***H***, *t*_(119)_ = 2.76; *p* = 0.0067) or Mann–Whitney rank sum tests [***F***, *U*(*n*_1_ = 35; *n*_2_ = 47) = 666; *p* = 0.1443; ***G***, *U*(*n*_1_ = 57; *n*_2_ = 64) = 1387; *p* = 0.0228].

**Figure 2.**
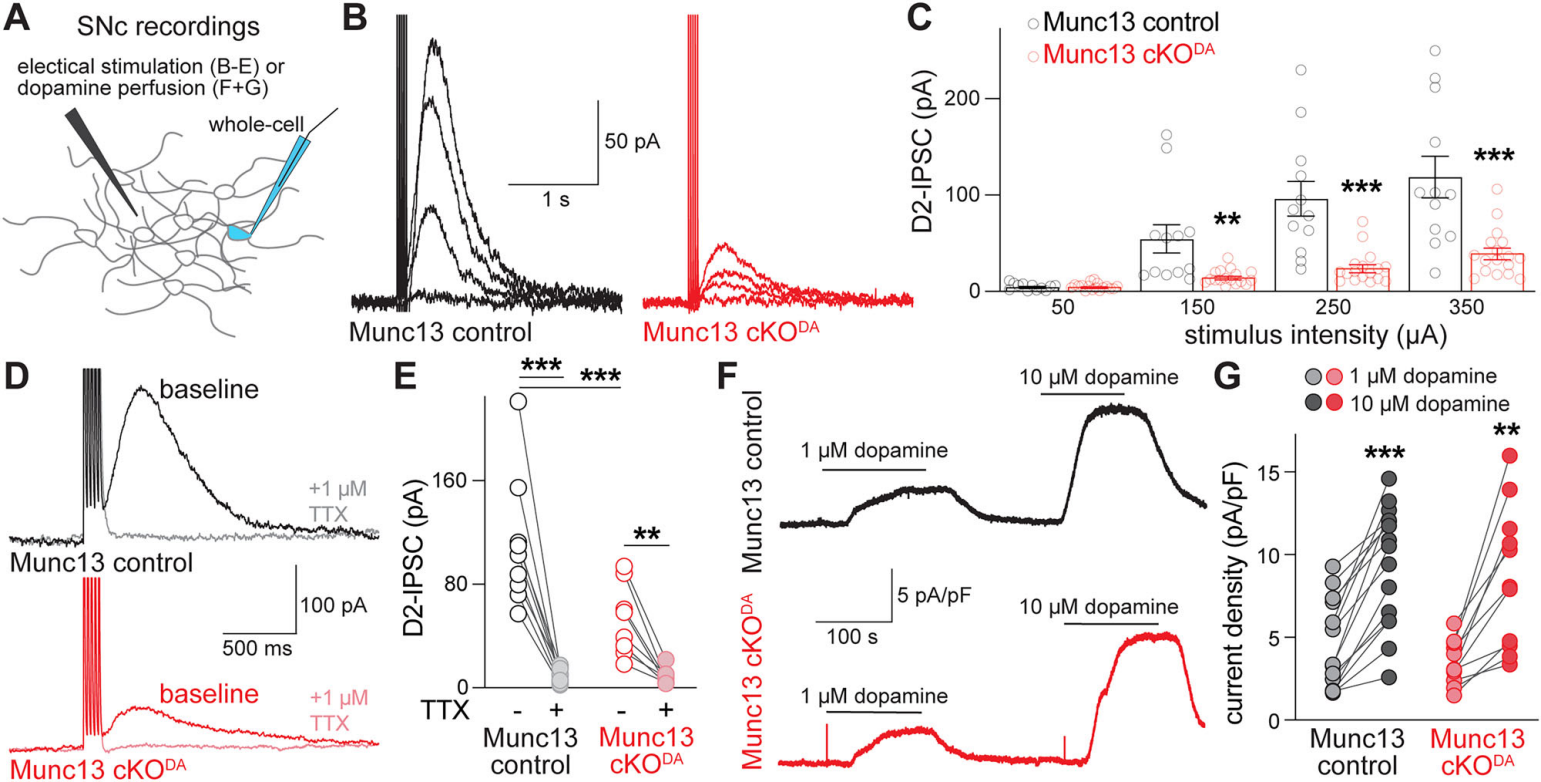
Munc13 ablation impairs dopamine release in the SNc. ***A***, Schematic of recordings in the SNc. ***B*, *C***, Example traces (***B***) and quantification of peak amplitudes (***C***) of D2-IPSCs induced by five stimuli at 40 Hz at 50, 150, 250, or 350 µA stimulation intensity; Munc13 control 12 cells from 10 slices of six mice, Munc13 cKO^DA^ 17/15/7. ***D*, *E***, Example traces (***D***) and quantification of peak amplitudes (***E***) of D2-IPSCs induced by five stimuli at 40 Hz at 350 µA stimulation intensity before or after perfusion of the voltage-gated Na^+^ channel blocker TTX (1 µm); Munc13 control 9/9/6, Munc13 cKO^DA^ 8/8/7. ***F*, *G***, Example traces (***F***) and quantification (***G***) of current density induced by 1 or 10 µM dopamine perfusion for ∼2 min until a peak current was achieved (calculated as the change in holding current divided by cell capacitance); Munc13 control 15/15/7, Munc13 cKO^DA^ 11/11/9. Data are shown as mean ± SEM; **p < 0.01; ***p < 0.001; assessed by two-way ANOVA in ***C*** (genotype, ***; intensity, ***; interaction, ***; *F*_Genotype(1,27)_ = 16.68; *p* = 0.0004; *F*_Intensity(3,81)_ = 53.11; *p* < 0.0001; *F*_Interaction(3,81)_ = 16.67; *p* < 0.0001), ***E*** (genotype, *; TTX, ***; interaction, **; *F*_Genotype(1,15)_ = 7.691; *p* = 0.0142; *F*_TTX(1,15)_ = 59.01; *p* < 0.0001; *F*_Interaction(1,15)_ = 9.201; *p* = 0.0084), and ***G*** (genotype, ns; concentration, ***; interaction, ns; *F*_Genotype(1,48)_ = 1.001; *p* = 0.3222; *F*_Concentration(1,48)_ = 31.81; *p* < 0.0001; *F*_Interaction(1,48)_ = 0.025; *p* = 0.8748) followed by Sidak's multiple-comparison tests (***C***, ***E***, ***G***, significance indicated in panels).

Roles of Munc13 in somatodendritic dopamine release were examined using whole-cell recordings from SNc dopamine neurons in horizontal slices of mice in which Munc13 proteins were genetically ablated. We pursued a strategy previously used to study axonal dopamine release (Banerjee et al., 2022) with dopamine neuron-specific ablation of Munc13-1 and constitutive knock-out of Munc13-2 and Munc13-3 (Munc13 cKO^DA^; Fig. 1*A*). In whole-cell recordings from dopamine neurons (Fig. 1*B*) of Munc13 cKO^DA^ and Munc13 control mice, the spontaneous firing rates and inward currents induced by a hyperpolarizing step (*I_H_*) were similar (Fig. 1*C*–*F*). The input resistance (*R_M_*) and cell capacitance of these neurons were also within expected ranges, but small magnitude decreases in *R_M_* and increases in capacitance were detected in slices of Munc13 cKO^DA^ mice (Fig. 1*G*,*H*). This is consistent with the modestly altered axon structure in the striatum of these mice (Banerjee et al., 2022). Midbrain dopamine transmission was measured via D2-IPSCs recorded in the SNc evoked by extracellular electrical stimulation (5 stimuli, 40 Hz) at varying intensities (Fig. 2*A*–*C*). At the lowest intensity (50 µA), neither Munc13 cKO^DA^ nor Munc13 control cells exhibited an appreciable D2-IPSC. As stimulus intensity increased, D2-IPSCs were induced in dopamine neurons of Munc13 control and Munc13 cKO^DA^ mice. D2-IPSC amplitudes in Munc13 cKO^DA^ slices were reduced by 73% at 150 µA stimulation intensity, 74% at 250 µA, and 67% at 350 µA. In both genotypes, D2-IPSCs were mediated by action potentials as they were eliminated by acute bath application of the Na^+^ channel blocker TTX (Fig. 2*D*,*E*).

We next tested whether the genetic manipulations might alter D2 receptor activation. We measured D2 receptor-mediated currents induced in response to perfusion of 1 or 10 µM exogenous dopamine (Fig. 2*F*,*G*). Quantification of the current density did not reveal a difference between Munc13 cKO^DA^ and control neurons. This establishes that somatodendritic D2 receptors are present in Munc13 cKO^DA^ neurons and that these receptors detect exogenous dopamine similar to the control condition. These observations support that the reduced D2-IPSC amplitudes are a result of decreased release from dopamine neurons following ablation of Munc13.

### Munc13 ablation similarly disrupts axonal and somatodendritic release

**Figure 3.**
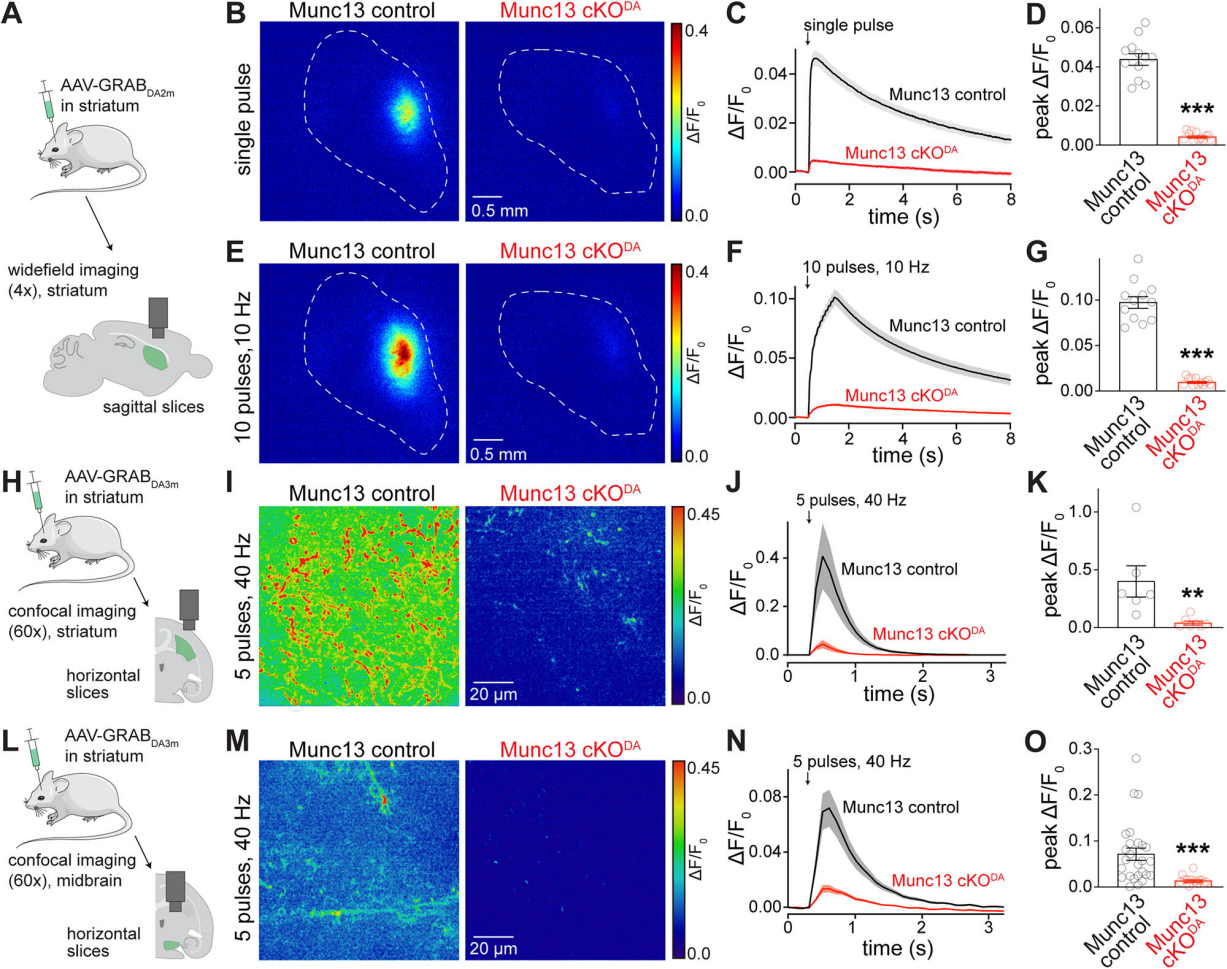
Munc13 ablation disrupts striatal and midbrain dopamine release monitored with GRAB_DA_. ***A***, Schematic of striatal unilateral AAV injections followed by widefield imaging in parasagittal striatal slices. ***B*–*D***, Example Δ*F*/*F*_0_ images (***B***) and quantification of the Δ*F*/*F*_0_ time course (***C***) and peak amplitude (***D***) during single extracellular electrical stimuli; Munc13 control 12 images of 12 slices from three mice, Munc13 cKO^DA^ 12/12/3. ***E*–*G***, As in ***B***–***D***, but for 10 stimuli at 10 Hz; Munc13 control 12/12/3, Munc13 cKO^DA^ 12/12/3. ***H***, Schematic of bilateral striatal AAV injections followed by confocal imaging in hemisected horizontal striatal slices. ***I****–**K***, Example Δ*F*/*F*_0_ images (***I***) and quantification of the Δ*F*/*F*_0_ time course (***J***) and peak amplitude (***K***) during five electrical stimuli at 40 Hz and 250 µA stimulation intensity; Munc13 control 6/4/4, Munc13 cKO^DA^ 7/3/3. ***L***, Schematic of bilateral striatal AAV injections followed by confocal imaging in hemisected horizontal midbrain slices. ***M****–**O***, As in ***I*–*K***, but for horizontal midbrain slices; Munc13 control 26/6/3, Munc13 cKO^DA^ 14/6/4. Data are shown as mean ± SEM; **p < 0.01; ***p < 0.001; assessed by unpaired *t* tests (***D***, *t*_(22)_ = 13.17; *p* < 0.0001; ***G***, *t*_(22)_ = 13.56; *p* < 0.0001) or Mann–Whitney rank sum test [***K***, *U*(*n*_1_ = 6; *n*_2_ = 7) = 1; *p* = 0.0023; ***O***, *U*(*n*_1_ = 26; *n*_2_ = 14) = 47; *p* < 0.0001].

A more direct measure of dopamine release was next obtained by imaging fluorescence changes of GPCR-activation based dopamine (GRAB_DA_) sensors. In a first set of imaging experiments, we used widefield microscopy as established before to assess axonal dopamine release in the striatum with the D2 receptor-based dopamine sensor GRAB_DA2m_ (Sun et al., 2020; Cai et al., 2024; Fig. 3*A*). GRAB_DA_ was expressed with AAVs injected into the striatum. Acute brain slices were prepared several weeks after injection, and GRAB_DA_ fluorescence changes were measured and quantified as Δ*F*/*F*_0_ in a large field of view. GRAB_DA_ fluorescence transients in response to either single stimuli or trains of 10 stimuli at 10 Hz (Fig. 3*B*–*G*) were strongly reduced in Munc13 cKO^DA^ slices. This confirms previous work that defined Munc13 proteins as important mediators of evoked axonal dopamine release with carbon fiber amperometry in acute striatal brain slices (Banerjee et al., 2022).

In a next set of experiments, we used a retrograde AAV strategy expressing GRAB_DA3m_, a D1 receptor-based dopamine sensor with an increased signal-to-noise ratio compared with GRAB_DA2m_ (Zhuo et al., 2024). We injected AAV-GRAB_DA_ into the striatum to transduce striatal neurons and neurons with afferent projections into the striatum. This strategy transduces dopamine neurons via their striatal axons. GRAB_DA_ fluorescence was then imaged using spinning-disc confocal microscopy in smaller fields of view compared with the widefield imaging experiments described above and with a stimulus protocol (5 stimuli, 40 Hz) well suited for midbrain measurements. We used this approach in parallel in the striatum and the midbrain to assess dopamine release in both areas (Fig. 3*H*,*L*). Similar to the widefield measurements (Fig. 3*A*–*G*), there was a robust impairment of dopamine release in Munc13 cKO^DA^ slices in the striatum (Fig. 3*H*–*K*). The assessment of stimulus-induced GRAB_DA_ transients in the ventral midbrain of Munc13 cKO^DA^ and Munc13 control slices revealed a strong reduction in somatodendritic dopamine release (Fig. 3*L*–*O*). Overall, the reduction in somatodendritic dopamine release monitored with GRAB_DA_ was similar in magnitude to effects observed in striatal GRAB_DA_ experiments (Fig. 3*A*–*K*) and in midbrain D2-IPSC recordings (Fig. 2*A*–*E*). Hence, axonal and somatodendritic dopamine release have a comparable dependency on Munc13. These findings are reminiscent of previous work that established shared dependency of axonal and somatodendritic dopamine release on RIM active-zone scaffolds and on the Ca^2+^ sensor Synaptotagmin-1 (Liu et al., 2018; Robinson et al., 2019; Banerjee et al., 2020, 2022; Delignat-Lavaud et al., 2023; Lebowitz et al., 2023).

### Spontaneous somatodendritic dopamine transmission persists after Munc13 ablation from dopamine neurons

**Figure 4.**
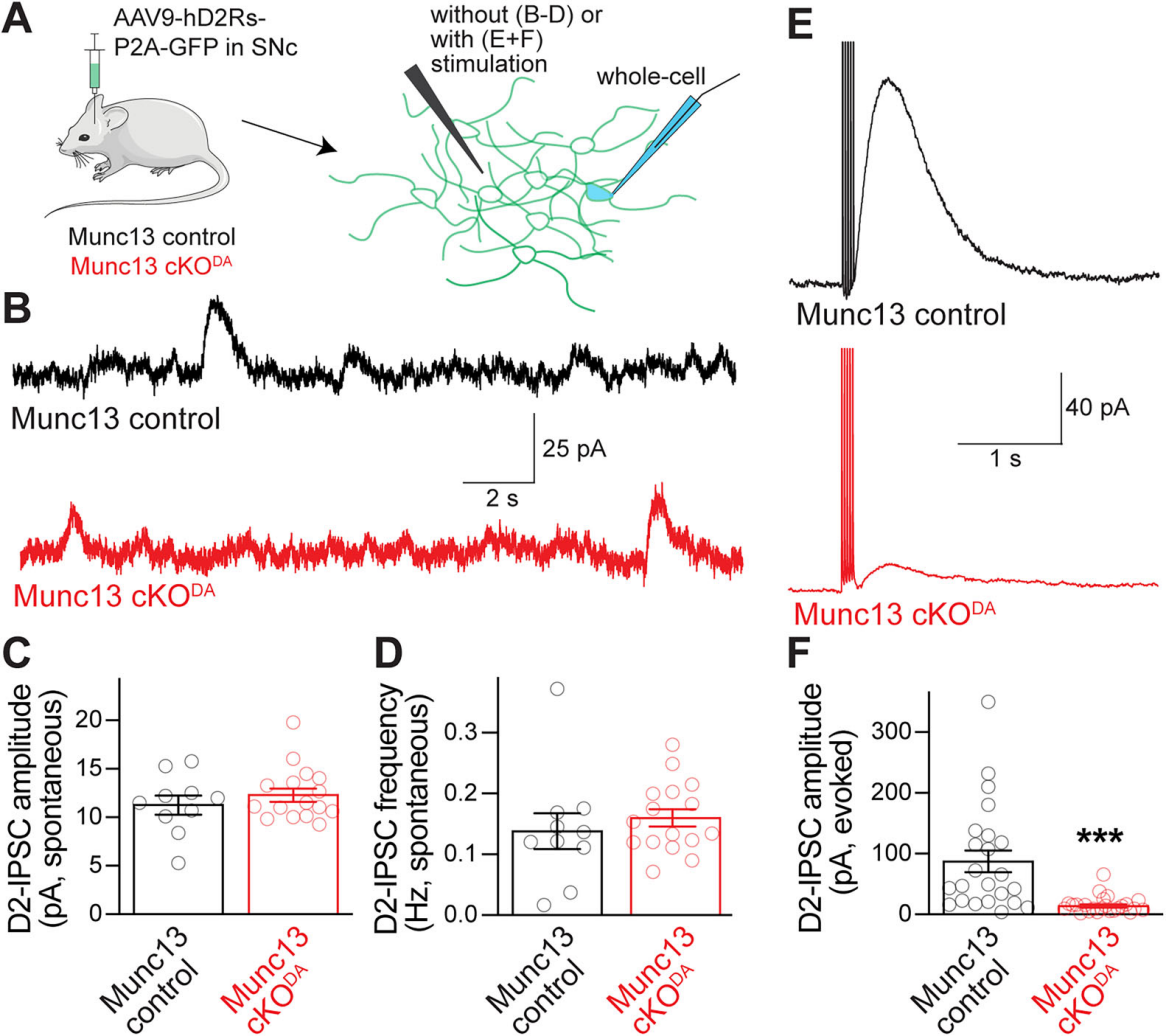
Spontaneous dopamine release in the SNc persists after Munc13 ablation. ***A***, Schematic of AAV-mediated D2 receptor expression followed by whole-cell recordings of SNc dopamine neurons. ***B*–*D***, Example traces (***B***) and quantification of amplitude (***C***) and frequency (***D***) of spontaneous D2-IPSCs; Munc13 control 10 cells from four slices of four mice, Munc13 cKO^DA^ 16/8/4. ***E*, *F***, Example traces (***D***) and quantification of peak amplitudes (***E***) of D2-IPSCs induced by five stimuli at 40 Hz and 150 µA stimulation intensity; Munc13 control 23/17/4, Munc13 cKO^DA^ 24/13/4. Data are shown as mean ± SEM, *** *p* < 0.001; assessed by Mann–Whitney rank sum tests (**C**: *U*(*n*_1_ = 10, *n*_2_ = 16) = 71, *p* = 0.6599, **F**: *U*(*n*_1_ = 23, *n*_2_ = 24) = 69, *p* < 0.0001) or an unpaired *t* test (**D**: *t*_(24)_ = 0.6937, *p* = 0.4945). For sensitivity of D2-IPSCs to sulpiride or Cd^2+^, see Figs. S1, S2.

Unlike evoked release, spontaneous somatodendritic dopamine release is insensitive to blockade of voltage-gated Na^+^ channels, voltage-gated Ca^2+^ channels, lowering of extracellular Ca^2+^, or genetic ablation of RIM or Synaptotagmin-1 from dopamine neurons (Beckstead et al., 2004; Gantz et al., 2013; Robinson et al., 2019; Lebowitz et al., 2023). Spontaneous glutamate or GABA release at fast synapses is greatly reduced when Munc13 is ablated (Varoqueaux et al., 2002; Tan et al., 2022a,b). We set out to examine whether spontaneous dopamine release shows a similar dependency on Munc13. We used a previously employed strategy to facilitate spontaneous D2-IPSC detection by exogenous expression of D2 receptors via AAVs injected into the midbrain (Fig. 4*A*; Lebowitz et al., 2023). We previously established that lowering extracellular Ca^2+^ or blockade of action potential firing with TTX does not decrease the frequency of the spontaneous release events in these experiments (Lebowitz et al., 2023). Here, we further established this methodology by assessing the sensitivity of spontaneous D2-IPSCs to D2 receptor blockade with sulpiride and voltage-gated Ca^2+^ channel blockade with Cd^2+^. In acute brain slices prepared ∼2 weeks after AAV transduction, bath addition of sulpiride abolished spontaneous and evoked D2-IPSCs (Fig. S1), establishing their D2 receptor dependence. In additional experiments, Cd^2+^ did not detectably inhibit spontaneous D2-IPSCs, supporting their independence of voltage-gated Ca^2+^ channels, but impaired evoked D2-IPSCs (Fig. S2). These results further establish the strategy of assessing spontaneous somatodendritic dopamine transmission in mice with AAV-mediated D2 receptor expression.

We then assessed spontaneous somatodendritic dopamine release using this experimental strategy in slices of Munc13 cKO^DA^ and Munc13 control mice (Fig. 4*A*). Neither the amplitude nor the frequency of spontaneous D2-IPSCs were changed in Munc13 cKO^DA^ slices (Fig. 4*B*–*D*). To assess whether the D2 receptor overexpression strategy altered the phenotypes observed in response to electrical stimulation, evoked D2-IPSCs were measured. Similar to the D2-IPSCs mediated by endogenous receptors (Fig. 2), the current amplitudes were strongly reduced after ablation of Munc13 in slices expressing exogenous D2 receptors (Fig. 4*E*,*F*). Hence, expression of exogenous D2 receptors did not alter the impairment in stimulated somatodendritic dopamine release. Previous work describing the independence of spontaneous release on RIM or Synaptotagmin-1, or on Ca^2+^ entry, is consistent with a model that spontaneous release in the midbrain occurs through a secretory pathway that is distinct from that for evoked release (Gantz et al., 2013; Robinson et al., 2019; Lebowitz et al., 2023). The lack of reliance on Munc13 further supports the model of a separate release pathway for spontaneous release in the midbrain.

### Norepinephrine innervation modestly contributes to D2-IPSCs measured in the midbrain

**Figure 5.**
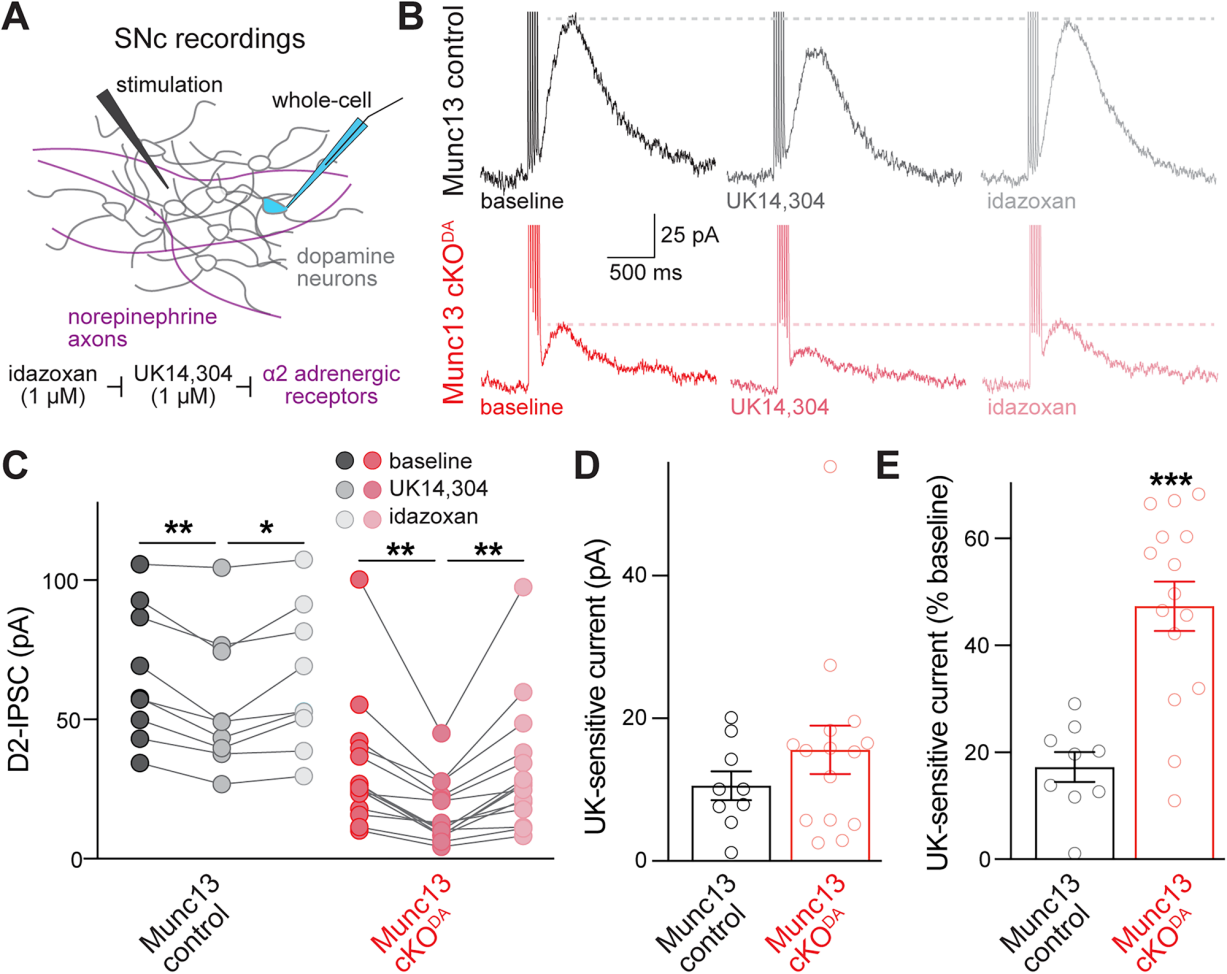
Contribution of release from norepinephrine axons to D2-IPSCs in the SNc. ***A***, Schematic and pharmacological strategy of D2-IPSC recordings in the SNc. ***B*–*E***, Example traces (***B***) and quantification of peak amplitudes (***C***) of D2-IPSCs induced by five stimuli at 40 Hz and 250 µA stimulation intensity, and quantification of absolute (***D***) and normalized (***E***) UK14,304 sensitivity in an experiment with sequential addition of UK14,304 and idazoxan; the dashed line in ***B*** marks baseline peak amplitude; Munc13 control nine cells from nine slices of four mice, Munc13 cKO^DA^ 15/15/6. Data are shown as individual cells (***C***) or as mean ± SEM; *p < 0.05; **p < 0.01; ***p < 0.001; assessed by a two-way ANOVA (***C***; genotype, ***; drug, ***; interaction, ns; *F*_Genotype(1,22)_ = 15.95; *p* = 0.0006; *F*_Drug(1.251,27.51)_ = 25.55; *p* < 0.0001; *F*_Interaction(1.251,27.51)_ = 1.698; *p* = 0.2053) followed by Tukey's post-tests (indicated in the figure), a Mann–Whitney rank sum test [***D***; *U*(*n*_1_ = 9; *n*_2_ = 15) = 53; *p* = 0.4115], or an unpaired *t* test (***E***; *t*_(22)_ = 4.734; *p* < 0.0001).

The residual component of dopamine release in Munc13 cKO^DA^ mice could be explained by Munc13 independence, by the presence of a small amount of a left-over Munc13 protein, or by neurotransmitters other than dopamine that might activate D2 receptors or dopamine sensors (Banerjee et al., 2022; Tan et al., 2022a,b; López et al., 2026). The substantia nigra receives norepinephrine innervation, and neurotransmitter released from these axons might act on D2 receptors (Mejías-Aponte et al., 2009; Agster et al., 2013; Sánchez-Soto et al., 2016; López et al., 2026). To examine this possibility, D2-IPSCs were measured before and after inhibition of release from norepinephrine axons by the selective α2-adrenergic receptor agonist UK14,304 (Fig. 5*A*). Activation of this *G_i_*-coupled autoreceptor is known to inhibit stimulation-evoked norepinephrine release (Hu and Fredholm, 1989; Rump et al., 1991; Bücheler et al., 2002). D2-IPSCs recorded in control slices showed a modest (17%) but statistically significant decrease in amplitude after bath addition of UK14,304, which was reversed by the α2 adrenergic receptor antagonist idazoxan (Fig. 5*B*–*E*). In neurons recorded in Munc13 cKO^DA^ slices, the remaining D2-IPSC was reduced by a similar absolute magnitude in response to bath application of UK14,304, though the smaller initial D2-IPSC amplitude before application of UK14,304 resulted in a larger relative inhibition (47%), which was also reversed by idazoxan (Fig. 5*B*–*E*). We conclude that only a small component of the D2-IPSC is mediated by norepinephrine axons. While this mechanism contributes significantly to the measured D2-IPSC that remains after Munc13 ablation, it does not fully account for it, and a small amount of somatodendritic dopamine release persists after the genetic ablation of Munc13 tested here.

## Discussion

The D2-IPSC and dopamine sensor measurements establish that the vesicle priming protein Munc13 is important for evoked somatodendritic dopamine release. Effect magnitudes of Munc13 ablation are similar for axonal and somatodendritic dopamine release. There is a small midbrain D2-IPSC remaining after Munc13 ablation, and about half of it is accounted for by release from norepinephrine axons. Contrasting evoked somatodendritic release, spontaneous D2-IPSCs are independent of Munc13 in dopamine neurons. Overall, our work is consistent with the model of preorganized sites for spatiotemporally precise somatodendritic dopamine release.

### Are there sites to prime vesicles for somatodendritic dopamine release?

For fast, action potential-induced secretion, fusion-ready vesicles must be pre-tethered at release sites (Neher, 2015; Kaeser and Regehr, 2017). In addition, voltage-gated Ca^2+^ channels need to be concentrated near the vesicular Ca^2+^ sensors (Eggermann et al., 2011; Kaeser and Regehr, 2014; Chin and Kaeser, 2024). At conventional synapses and in dopamine axons, active-zone proteins orchestrate these processes (Südhof, 2013; Liu and Kaeser, 2019).

Whether similar protein assemblies control somatodendritic neurotransmitter secretion has remained an open question. Munc13's importance provides molecular evidence for vesicle priming in midbrain dopamine secretion. Munc13 is the principal priming protein at synapses, where it mediates the assembly of SNARE complexes (Augustin et al., 1999; Varoqueaux et al., 2002; Dulubova et al., 2005; Ma et al., 2011; Lai et al., 2017) and supports the tight docking of synaptic vesicles (Siksou et al., 2009; Imig et al., 2014; Tan et al., 2022a,b). Central to these roles is the recruitment and activation of Munc13 by RIM (Betz et al., 2001; Dulubova et al., 2005; Deng et al., 2011; Camacho et al., 2017; Tan et al., 2022a,b).

The findings that Munc13 (Figs. 2, 3) and RIM (Robinson et al., 2019) are important for stimulus-induced somatodendritic dopamine release support that the synaptic vesicle priming mechanism operates in somatodendritic dopamine secretion. More broadly, the dependence on Munc13, RIM (Robinson et al., 2019), and the vesicular Ca^2+^ sensor Synaptotagmin-1 (Lebowitz et al., 2023) suggests that somatodendritic secretion occurs at preassembled exocytic sites at the plasma membrane of somata and/or dendrites and imply that the vesicles are docked before fusion. While it has remained difficult to morphologically define somatodendritic release sites, the data presented here and in previous studies provide a foundation to identify them via the presence of RIM, Munc13, and Synaptotagmin-1. This is a critical goal, because axon collaterals might contribute to midbrain dopamine release, though their abundance in the substantia nigra is likely low (Wassef et al., 1981; Groves and Linder, 1983). Because RIM, Munc13, and Synaptotagmin-1 typically mediate the fusion of synaptic vesicles (Südhof, 2013; Kaeser and Regehr, 2014; Biederer et al., 2017), their roles in midbrain dopamine transmission also suggest that somatodendritic release is mediated by small, clear vesicles. This model differs from early studies that suggested fusion of tubulovesicular structures for somatodendritic release and is more consistent with reports on the presence of small, pleiomorphic vesicles (Hajdu et al., 1973; Wilson et al., 1977; Groves and Linder, 1983; Nirenberg et al., 1996). Overall, this and previous work identifies molecules important for midbrain dopamine release.

### Organization of somatodendritic transmission beyond vesicle priming

Many features of somatodendritic transmission remain understudied. Previous work on evoked release indicates tight coupling of Ca^2+^ entry and secretion with a high initial release probability, and complete Cd^2+^ sensitivity established the dependence on voltage-gated Ca^2+^ channels (Beckstead et al., 2004, 2007; Ford et al., 2010; Chen et al., 2011). The exact Ca^2+^ sources and mechanisms for their targeting to release sites, however, remain uncertain, and blocking L-, N- or P/Q-type channels inhibited somatodendritic only release partially (Beckstead et al., 2004; Chen et al., 2006; Liu et al., 2021). Deletion of the fast Ca^2+^ sensor Synaptotagmin-1 strongly impairs single pulse D2-IPSCs, but stimulus trains evoke significant somatodendritic release in Synaptotagmin-1 knock-out dopamine neurons (Lebowitz et al., 2023). Knockdown, knock-out, and antibody application experiments have also implicated Synaptotagmin-4 and Synaptotagmin-7 (Mendez et al., 2011; Delignat-Lavaud et al., 2022; Hikima et al., 2022; Lebowitz et al., 2024). While vertebrate Synaptotagmin-4 is unlikely to mediate Ca^2+^ sensing (Dai et al., 2004), effects of Synaptotagmin-7 ablation or inhibition are generally mild. Overall, the full complement of Ca^2+^ sensors for somatodendritic dopamine release remains unknown.

A determinant for efficacy of signaling is the positioning of receptors relative to release sites. At synapses, tight coupling at tens of nanometers controls receptor activation (Biederer et al., 2017). For somatodendritic dopamine transmission, release-receptor organization remains unclear. Morphological studies have identified discrete D2 receptor puncta (Gantz et al., 2015; Robinson et al., 2017; Lebowitz et al., 2022), but it has not been possible to assess their proximity to release sites. Functional imaging has established that somatodendritic dopamine release generates hotspots and that uncaging a D2 receptor antagonist inhibits receptor activation within the first ∼100 ms of stimulation, but not at later time points (Condon et al., 2021). In addition, D2-IPSC induction relies on a high dopamine concentration, 30–100 µM (Ford et al., 2009). These studies suggest that dopamine acts rapidly and in a contained space. It is noteworthy, though, that dopamine diffusion is fast, and receptors several micrometers away from release sites might be reached within 100 ms (Rice and Cragg, 2008; Yee et al., 2025). Future studies should assess release-receptor organization in the midbrain dopamine system to define dopamine's signaling mechanisms.

### Norepinephrine contribution to somatodendritic dopamine signaling

Norepinephrine neurons innervate the midbrain (Liprando et al., 2004; Mejías-Aponte et al., 2009; Mejias-Aponte, 2016), and α1 adrenoreceptors inhibit dopamine neuron activity (Paladini and Williams, 2004). Norepinephrine may also act as an agonist of D2 and D2-like receptors (White and Wang, 1984; Sánchez-Soto et al., 2016), and it activates D2 receptor–based dopamine sensors (López et al., 2026). Here, we find that norepinephrine innervation can contribute to D2-IPSCs. In control mice, the contribution is small (Fig. 5). In Munc13 cKO^DA^ mice, norepinephrine innervation accounts for half of the remaining D2-IPSC. In our experiments, whether released norepinephrine activates D2 receptors or whether norepinephrine neurons corelease dopamine before its conversion to norepinephrine (Weinshilboum et al., 1971; Stewart and Klinman, 1988; Özçete et al., 2024) remains unclear.

### Spontaneous somatodendritic dopamine release

Dopamine is associated with movement initiation and vigor, though its mechanisms remain debated (Carlsson, 1960; Panigrahi et al., 2015; Howe and Dombeck, 2016; Berke, 2018; da Silva et al., 2018; Cai et al., 2024; Liu et al., 2025). Recent studies suggest that spontaneous dopamine release suffices to mediate movement. In mice with disrupted evoked dopamine release and in vivo dopamine dynamics (via RIM ablation and/or pharmacological manipulations), movement initiations persist (Cai et al., 2024). Spontaneous somatodendritic dopamine release is unaffected by RIM ablation (Robinson et al., 2019). Our findings bolster the model that evoked dopamine release is dispensable for movement. Munc13 cKO^DA^ mice have a large reduction in evoked dopamine release (Figs. 2, 3). While their body weight at weaning is reduced, they survive, move, and eat (Banerjee et al., 2022).

The cellular pathway for spontaneous somatodendritic dopamine release remains to be defined. Previous work indicates that it is vesicular and reveals independence of voltage-gated Na^+^ channels, voltage-gated Ca^2+^ channels, extracellular Ca^2+^, intracellular Ca^2+^ stores, and Synaptotagmin-1 and RIM expression in dopamine neurons (Gantz et al., 2013; Robinson et al., 2019; Lebowitz et al., 2023). Here, we show that spontaneous somatodendritic dopamine transmission persists after Munc13 ablation (Fig. 4). The release pathway may not only be different from that of evoked release, but it might also differ from that of spontaneous release at synapses, where ablations of RIM, Munc13, or Synaptotagmin-1 alter event frequencies across synapses, cell types, and species (Varoqueaux et al., 2002; Yoshihara and Littleton, 2002; Xu et al., 2009; Deng et al., 2011; Zhou et al., 2015; Tan et al., 2022a,b). What could those pathways be? One possibility is that distinct SNARE proteins or their regulators may control spontaneous dopamine release. For example, Vti1a, Vamp7, and Doc2 have been implicated in spontaneous fusion at synapses (Groffen et al., 2010; Hua et al., 2011; Ramirez et al., 2012; Kaeser and Regehr, 2014), and they may have roles in controlling spontaneous fusion events in dopamine neurons. Furthermore, recent work has identified adaptor complexes for vesicles released during high-frequency firing of dopamine neurons (Jain et al., 2023). Similar mechanisms could help generate a pool of vesicles for spontaneous release. Alternatively, spontaneous dopamine release may be mediated by a different secretory pathway, by different cells, or by a small amount of Munc13 present in the tested mutants (Banerjee et al., 2022). Regarding an alternate pathway, the exocyst is a candidate that consists of conserved secretory machinery, though it typically participates in neuronal development but not transmitter exocytosis (Murthy et al., 2003; He and Guo, 2009).

Overall, our studies provide a molecular handle on the identity of the sites that underlie evoked release in the midbrain, as they indicate that Munc13 might be a marker similar to synaptic release sites (Reddy-Alla et al., 2017; Sakamoto et al., 2018). The alternate pathways described above should be systematically investigated for a role in spontaneous somatodendritic dopamine release.
